# Hepatic Fgf21 Expression Is Repressed after Simvastatin Treatment in Mice

**DOI:** 10.1371/journal.pone.0162024

**Published:** 2016-09-01

**Authors:** Panos Ziros, Zoi Zagoriti, George Lagoumintzis, Venetsana Kyriazopoulou, Ralitsa P. Iskrenova, Evagelia I. Habeos, Gerasimos P. Sykiotis, Dionysios V. Chartoumpekis, Ioannis G Habeos

**Affiliations:** 1 Department of Internal Medicine, Division of Endocrinology, School of Medicine, University of Patras, Patras, Greece; 2 Department of Pharmacy, University of Patras, Patras, Greece; Lady Davis Institute for Medical Research, CANADA

## Abstract

Fibroblast growth factor 21 (Fgf21) is a hormone with emerging beneficial roles in glucose and lipid homeostasis. The interest in Fgf21 as a potential antidiabetic drug and the factors that regulate its production and secretion is growing. Statins are the most widely prescribed drug for the treatment of dyslipidemia. However, the function of statins is not limited to the lowering of cholesterol as they are associated with pleiotropic actions such as antioxidant, anti-inflammatory and cytoprotective effects. The recently described effect of statins on mitochondrial function and the induction of Fgf21 by mitochondrial stress prompted us to investigate the effect of statin treatment on Fgf21 expression in the liver. To this end, C57BL6J male mice and primary mouse hepatocytes were treated with simvastatin, and Fgf21 expression was subsequently assessed by immunoblotting and quantitative real-time PCR. Hepatic Fgf21 protein and mRNA and circulating levels of FGF21significantly decreased in mice that had received simvastatin in their food (0.1% w/w) for 1 week. This effect was also observed with simvastatin doses as low as 0.01% w/w for 1 week or following 2 intraperitoneal injections within a single day. The reduction in Fgf21 mRNA levels was further verified in primary mouse hepatocytes, indicating that the effect of simvastatin is cell autonomous. In conclusion, simvastatin treatment reduced the circulating and hepatic Fgf21 levels and this effect warrants further investigation with reference to its role in metabolism.

## Introduction

Fibroblast Growth Factor 21 (Fgf21) is a hormone that exerts beneficial effects on various metabolic parameters, such as glucose and lipid homeostasis and weight loss. These features make it a promising potential therapy for the treatment of type 2 diabetes [1–3]. Fgf21 is primarily expressed in the liver, white and brown adipose tissues, and the pancreas [4, 5]. However, circulating levels of FGF21 are derived primarily, if not exclusively, from the liver [6].

FGF21 signals to other tissues through a receptor complex consisting of fibroblast growth factor receptor 1c (Ffgr1c) and β-klotho. The restricted expression pattern of β-klotho mediates the tissue-specific functions of Fgf21 in various tissues and organs, including adipose tissue, the pancreas and the hypothalamus [7, 8].

Endogenous Fgf21 plays an important role in energy homeostasis and adaptation to stressful conditions, such as nutrient starvation and overfeeding. Its function is also influenced by the presence or absence of other endocrine signals. Fgf21 regulates energy homeostasis under conditions of fluctuating nutrient availability. During fasting, Fgf21 regulates hepatic gluconeogenesis, beta oxidation [9] and adipose tissue lipolysis [10]. During refeeding, Fgf21 enhances insulin-stimulated glucose uptake [6]. Circulating levels of Fgf21 are elevated in response to overfeeding in order to mitigate decreasing insulin sensitivity and facilitate the disposal of glucose to brown adipose tissue [6]. In addition, serum Fgf21 levels are increased in diet-induced obese mice that respond poorly to exogenous Fgf21 administration, indicating that these mice developed a resistance to Fgf21 [11].

In addition to nutritional stress[10–12], expression of Fgf21 is affected by amino acid deprivation [13], chemicals such as acetaminophen or dioxin [14, 15], cold conditions[16–18], oxidative stress, mitochondrial stress[19–21] and ER stress[22–24]. Recently, a deficiency in autophagy was also linked to the activation of Fgf21 [21, 25].

Fgf21 is transcriptionally regulated by peroxisome proliferator-activated receptor alpha (Pparα)[10, 26], hepatocyte specific cAMP-responsive element binding protein (Crebh) [27], peroxisome proliferator-activated receptor γ (Pparγ)[28, 29] and other factors. Pparγ agonists (thiazolidinediones)[30, 31], glucagon-like peptide-1 analogs[32] and metformin[20], agents used for the treatment of diabetes, exert their actions at least partially through Fgf21.

Statins are inhibitors of 3-hydroxy-3-methylglutarylcoenzyme A (HMG-CoA) reductase, the rate-limiting enzyme in the mevalonate pathway. This pathway produces cholesterol, as well as coenzyme Q (a component of the respiratory chain), dolichols (compounds important for protein glycosylation) and isoprenoids (lipid moieties responsible for the membrane association of small GTPases). In addition to reducing LDL cholesterol by activating SREBP2[33], statins have pleotropic actions such as antioxidant and anti-inflammatory effects that contribute to cardio- and neuro-protection[34–37]. Some of these pleotropic effects have been attributed to the blockade of other branches of the mevalonate pathway.

Impairment of isoprenoid synthesis by statins affects mitochondrial function in C. elegans and in mammalian cells [38–41]. Recent studies have even linked mevalonate blockade by statins to mitochondrial dysfunction and autophagy [42–44]. Moreover, the inhibition of protein prenylation by statins can induce the ER stress response [45–47]. As Fgf21 is induced by several types of mitochondrial stress and statins potentially affect mitochondrial function, it is reasonable to hypothesize that statins might affect Fgf21 expression. In the present study, we investigated the effect of statins and specifically simvastatin on Fgf21 expression in mice and primary hepatocytes.

## Materials and Methods

### Chemicals

Chemicals were purchased from Sigma (St Louis, MO) unless otherwise indicated.

Simvastatin was a kind gift from Vianex SA (Athens, Greece).

Simvastatin activation for the intraperitoneal (IP) injections of mice or for cell culture treatment was done according to a previously described method [48].

### Mice

C57BL6J wild-type (WT) male mice (Jackson Laboratories, Bar Harbor, ME) were housed in light- and humidity-controlled rooms in the animal facility of the School of Medicine of the University of Patras with a 12 h light/dark cycle, and they had free access to water. Mice were treated with simvastatin either by the ingestion of chow supplemented with 0%, 0.01%, 0.05%, 0.1% or 0.5% w/w simvastatin for 7 days or by 2 intraperitoneal (IP) injections of activated simvastatin at a dose of 15 mg/kg at 20 hours and 12 hours before sacrifice as indicated in the relevant figures. Mice were sacrificed by administering isoflurane anesthesia after 12 h fast. Animal experiments were approved by the relevant committee of the University of Patras, Greece (11/2011).

### Primary Mouse Hepatocyte Culture

Three-month old WT male mice were perfused through the heart with 5 mM EGTA in HBSS (Life technologies, Carlsbad, CA) and were subsequently perfused with 5 mM CaCl2/0.05% collagenase A (Roche, Indianapolis, IN) in HBSS. Hepatocytes were plated in collagen-treated 10 cm plates in DMEM supplemented with 10% fetal bovine serum (FBS) and penicillin/streptomycin (p/s) and incubated for 4 h at 37°C in a 5% CO2 incubator [49]. Following the incubation, the medium was changed, and the cells were treated with the indicated doses of simvastatin for the indicated time. For the primary mouse hepatocytes transfection experiments, the Viromer red (Lipocalyx, Germany) transfection reagent was used.

### HepG2 Culture and Transfection or Transduction

HepG2, a human hepatocellular carcinoma line, was obtained from ECACC (Salisbury,UK) and was maintained in DMEM supplemented with 10% FBS and p/s. Lipofectamine (Invitrogen) was used for the transfection of the Srebp-2 plasmid (pcDNA3.1-2xFLAG-SREBP-2, gift from Timothy Osborne, Addgene plasmid # 26807) [50] or the mock vector. The pAdTrack and pAdTrack-miR33 adenoviral particles were obtained from the Ángel Baldán lab [51] and were used to overexpress miR33 in HepG2 cells.

### Protein Extracts and Immunoblotting Analysis

Total protein extracts were prepared by lysing mouse livers in ice cold RIPA buffer as previously described[52] and used for protein electrophoresis and immunoblotting. The following primary antibodies were used: Ampka (#2532) and Elf2a (#9722) from Cell Signaling (Danvers, MA) and Fgf21(ab171941) from Abcam (Cambridge, UK). Densitometry analysis of the bands was performed on scanned X-ray films using the Image Studio Lite version 5.0 software (LI-COR Biosciences, Lincoln, NE).

### RNA Isolation and qRT-PCR

RNA from livers or from primary hepatocytes was prepared using the Trizol (Life Technologies) method. The RNA was subsequently purified using the RNAeasy kit (Qiagen, Hilden, Germany) on DNAse digestion columns to eliminate potential genomic DNA contamination as indicated by the manufacturer. RNA was quantified by spectrophotometry at 260 nm and purity was assessed by calculating the ratio of absorbance measurements (A_260nm_/A_280nm_). RNA samples were assessed for integrity by electrophoresis on a denaturing 1.2% agarose gel.

cDNA was synthesized from RNA using the PrimeScript RT Reagent Kit (Takara, Shiga, Japan). Real-time PCR was performed on a Step One Plus cycler (Applied Biosystems) using the KAPA SYBR FAST mix (KAPA Biosystems, Wilmington, MA) in triplicate 20 μl reactions. The PCR efficiency was calculated by the standard curve method, and the Pfaffl method was used to calculate fold-changes in expression levels[53]. The specificity of the PCR product was verified by melting curve analysis and by agarose gel electrophoresis. The expression of the Peptidylprolyl isomerase A (*Ppia*) gene was not affected by simvastatin treatment and was therefore used as a reference gene to normalize gene expression levels in the mouse liver and primary hepatocyte samples. TATA-box binding protein (*Tbp*) was employed as a reference gene to normalize gene expression levels in the HepG2 samples. The sequences of the primers used for qRT-PCR are shown in Table 1.

**Table 1:** Primer sequences used for qRT-PCR.

Gene	Forward	Reverse
*mPpia*	CAGACGCCACTGTCGCTTT	TGTCTTTGGAACTTTGTCTGCAA
*mFgf21*	CAAGACACTGAAGCCCACCT	CACCCAGGATTTGAATGACC
*mPcsk9*	ACCCTCATAGGCCTGGAGTT	CTGTGATGACCTCTGGAGCA
*mHmgcr*	CCCTGAGTTTAGCCTTCCTTTTG	GCTTTCTTTGAGGTCACGACGG
*mAcox1*	TCGAAGCCAGCGTTACGAG	ATCTCCGTCTGGGCGTAGG
*mCyp4a10*	GTGCTGAGGTGGACACATTCAT	TGTGGCCAGAGCATAGAAGATC
*mCyp7a1*	GCTGTGGTAGTGAGCTGTTG	AGGAGGTTCACCTACTTTCCTT
*hFGF21*	CTGTGGGTTTCTGTGCTGG	CCGGCTTCAAGGCTTTCAG
*hTBP*	ATGACTCCCGGAATCCCTATC	AGTGCCATAAGGCATCATTGG
*hABCA1*	CCCAGAGCAAAAAGCGACTC	GGTCATCATCACTTTGGTCCTTG
*hPCSK9*	ATGGTCACCGACTTCGAGAAT	GTGCCATGACTGTCACACTTG

### Measurement of Serum Fgf21

Serum Fgf21 levels were measured using an ELISA kit (MF2100) from R&D Biosystems (Minneapolis, MN) with the serum of mice that had been starved overnight.

### Statistical Analysis

Student’s t-test or one-way ANOVA followed by Tukey’s test was performed using GraphPad Prism 5 (GraphPad Software, La Jolla, CA). The number of subjects or replicates in each experiment is indicated in the relevant figure legends. Data are expressed as the mean ± SEM. P<0.05 was considered statistically significant.

## Results

### Fgf21 Protein and mRNA Levels in the Liver Decreased after Treatment with Simvastatin

**Fig 1:**
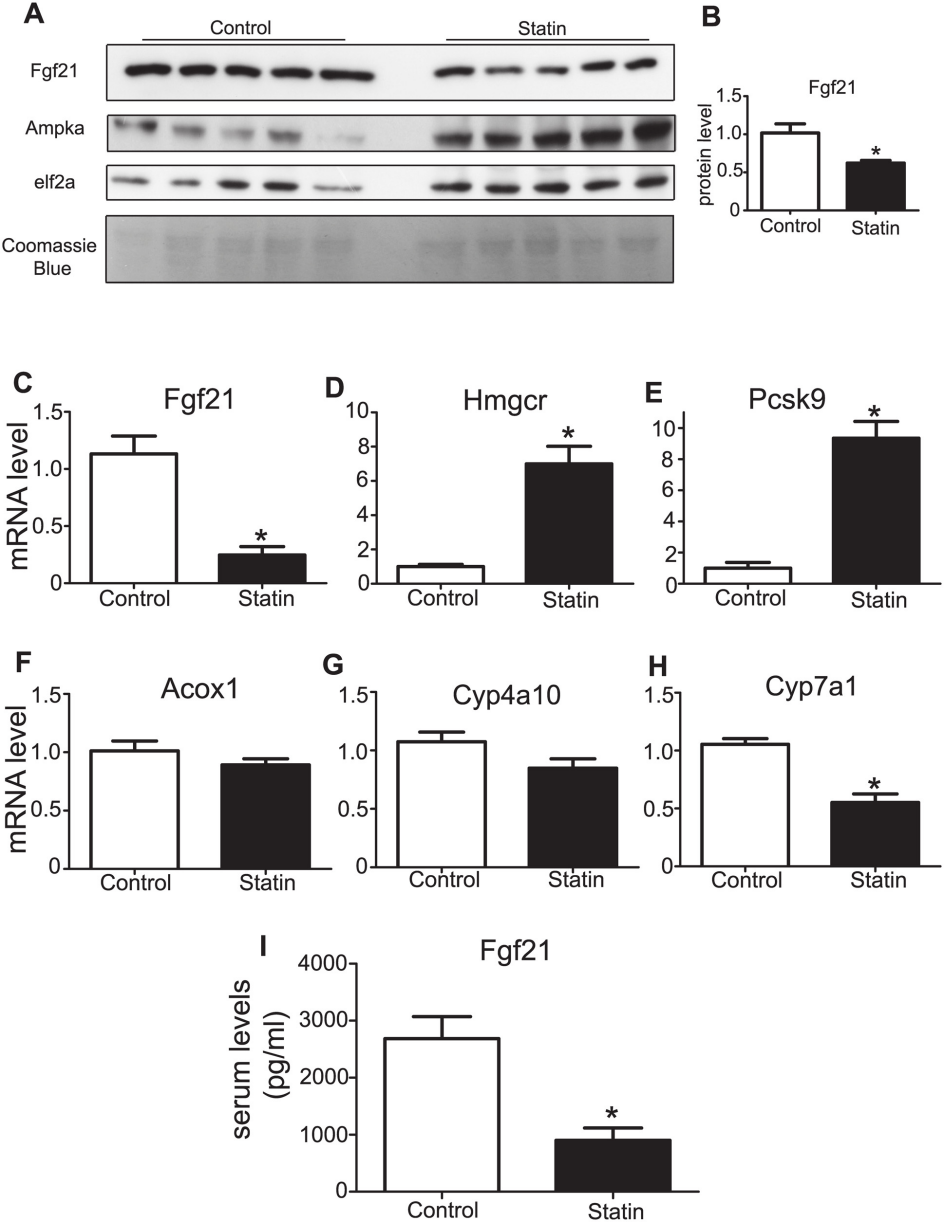
Expression of Fgf21 in liver and serum after simvastatin treatment in mice. **A.** Immunoblotting analysis of Fgf21 in the livers of 3-month old male mice treated with simvastatin (0.1% w/w in chow) for 1 week. Immunoblots for Ampkα and elf2a and Coomassie blue staining of the gel were used as loading controls. **B.** Relative Fgf21 protein levels as assessed by immunoblotting after normalization to elf2a levels. Data are presented as the mean ± SEM. n = 5 per treatment. *P<0.05. **C, D, E, F, G, H**. mRNA levels of Fgf21(C), Hmgcr (D), Pcsk9 (E), Acox1 (F), Cyp4a10 (G), Cyp7a1 (H) in the livers of 3-month old male mice treated with simvastatin (0.1% w/w in chow) for 1 week. Relative mRNA levels were assessed by qRT-PCR. Data are presented as the mean ±SEM. n = 7–8 per treatment. *P<0.05. **I.** Assessment of serum Fgf21 levels by ELISA in 3-month old male mice treated with vehicle or simvastatin. Data are presented as the mean ±SEM. n = 7–8 per treatment. *P<0.05.

Male WT mice treated with simvastatin (0.1% w/w in mouse chow) for 1 week exhibited decreased hepatic Fgf21 protein levels (Fig 1A). Fgf21 levels in the treated mice were reduced by approximately 50% compared with the control (Fig 1B). Consistent with the reduction in Fgf21 protein levels, hepatic Fgf21 mRNA levels in simvastatin-treated mice were also reduced (Fig 1C). Genes known to be upregulated by statin treatment, such as 3-Hydroxy-3-Methylglutaryl-CoA Reductase (*Hmgcr*) and Proprotein Convertase Subtilisin/Kexin Type 9 (*Pcsk9*), were employed as positive controls to verify the efficacy of simvastatin treatment. As expected, Hmgcr and Pcsk9 mRNA levels (Fig 1D and 1E) were upregulated approximately 7- and 9-fold, respectively. The expression of some of known Pparα-regulated genes (Acox1, Cyp4a10, Cyp7a1) was also examined (Fig 1F, 1G and 1H) as Pparα has been described to be one of the important regulators of Fgf21 expression [54, 55]. Only the expression of Cyp7a1 was found to be downregulated by almost 50% in the livers of simvastatin-treated mice.

### Circulating Fgf21 Levels Decreased in Mice Treated with Simvastatin

To confirm that serum levels of Fgf21 reflected the decrease in hepatic Fgf21 expression observed in simvastatin-treated mice, serum samples were collected from control and simvastatin-treated mice (0.1% w/w simvastatin in chow for 1 week) following an overnight 12 h fast. Fgf21 serum levels were measured using ELISA. Fgf21 serum level in the control mice was 2684 ± 388.5 pg/ml and 900.1 ± 219.1 pg/ml in simvastatin-treated mice (Fig 1I). This difference was statistically significant (P<0.05).

### Simvastatin Decreased Hepatic Fgf21 Levels in a Dose-Dependent Manner

**Fig 2:**
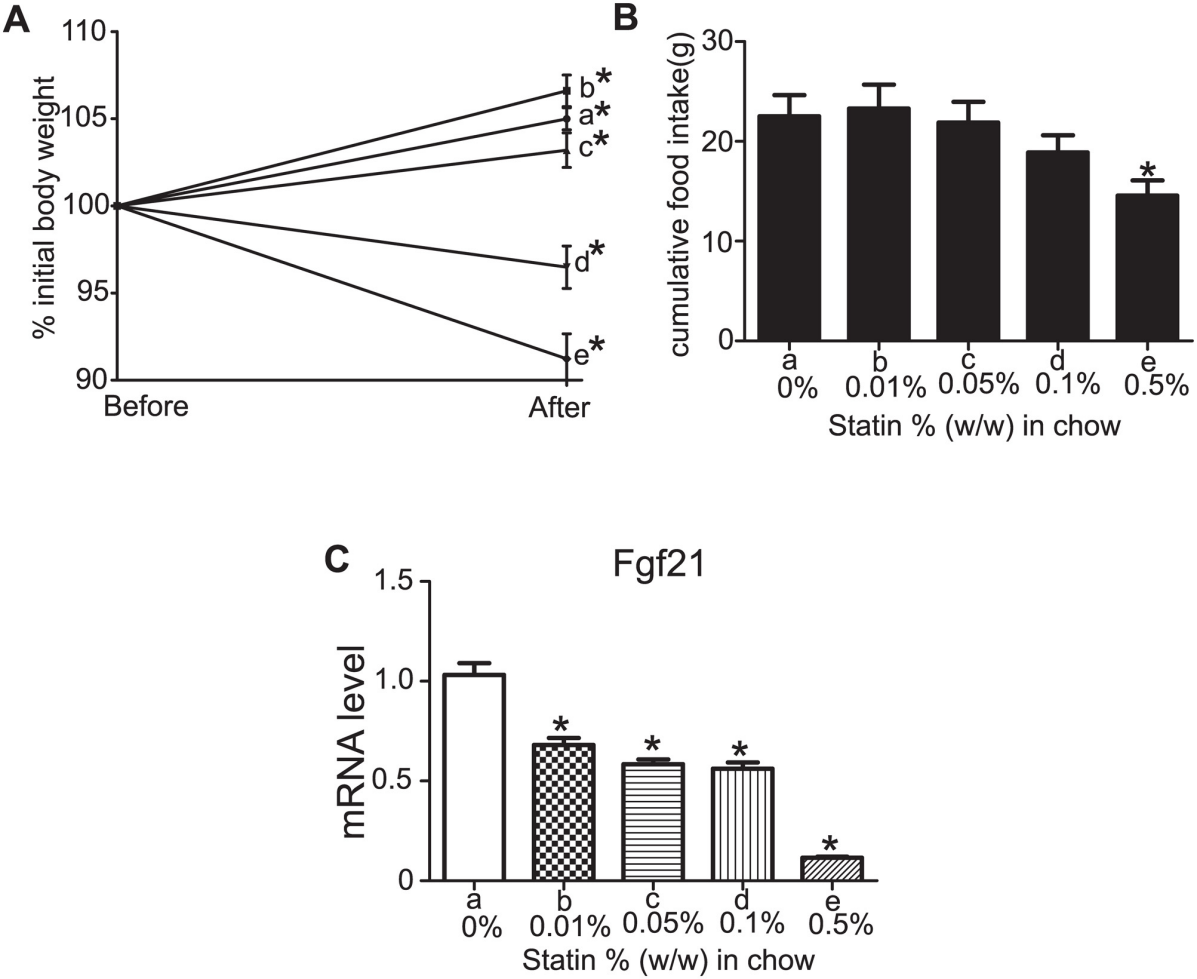
Body weights, food consumption and hepatic Fgf21 mRNA levels in 1-month old male mice following the administration of increasing doses of simvastatin for 1 week. **A.** Body weights of mice before and after exposure to simvastatin expressed as the % of initial weight. *P<0.05 compared with baseline weight. **B.** Cumulative food intake for the 1-week treatment with simvastatin. *P<0.05 compared with vehicle treatment (0% simvastatin w/w). **C.** Fgf21 hepatic mRNA levels as assessed by qRT-PCR. *P<0.05 compared with the 0% dose. a, b, c, d, e denote 0%, 0.01%, 0.05%, 0.1% and 0.5% w/w simvastatin in chow, respectively. For panels A, B, C the data are presented as the mean ± SEM. n = 6 per treatment with the exception of the 0.5% dose (n = 3; 5 mice received the treatment and 2 died after the treatment).

The dose of simvastatin (0.1% w/w in chow) used in the experiments described in the previous sections is the dose commonly used in other reported studies [56, 57]. To evaluate simvastatin-induced Fgf21 reduction over a wide range of doses and to assess potential toxicity, we evaluated the effect of a range of doses of simvastatin (0.01%, 0.05%, 0.1% and 0.5%). One-month-old male mice were treated with the aforementioned doses for 1 week and were sacrificed after an overnight 12 h fast. During the week of treatment, the mice were monitored daily for their well-being, and their body weights were measured after a 12h fast at the beginning and at the end of the study. The body weight of mice in the control group and the simvastatin-treated group with the doses of 0.01% and 0.05% increased after the week of treatment, as expected. However, the group treated with the highest dose of simvastatin (0.5%) lost 10% of their body weight and 2 mice in this group died on the last day of the treatment. Mice treated with 0.1% simvastatin also lost weight (3.5% reduction from the original body weight), but no deaths occurred and no change in the well-being of these mice was observed (Fig 2A). The food intake of these mice was also recorded and was found that in the 0.5% w/w simvastatin group the mice consumed significantly less chow than the control group (Fig 2B). The 0.1% group showed also a tendency for decreased chow consumption whereas no difference in chow consumption was seen in the other groups compared to control.

Assessment of mRNA levels using qRT-PCR revealed that all doses of simvastatin lowered hepatic Fgf21 mRNA levels (Fig 2C). The % reduction in Fgf21 expression from baseline was 32%, 42%, 44% and 88% in mice treated with 0.01%, 0.05%, 0.1% and 0.5% simvastatin, respectively, and these reductions were all statistically significant.

### Short-Term Simvastatin Administration Decreased Fgf21 Levels in the Mouse Liver

**Fig 3:**
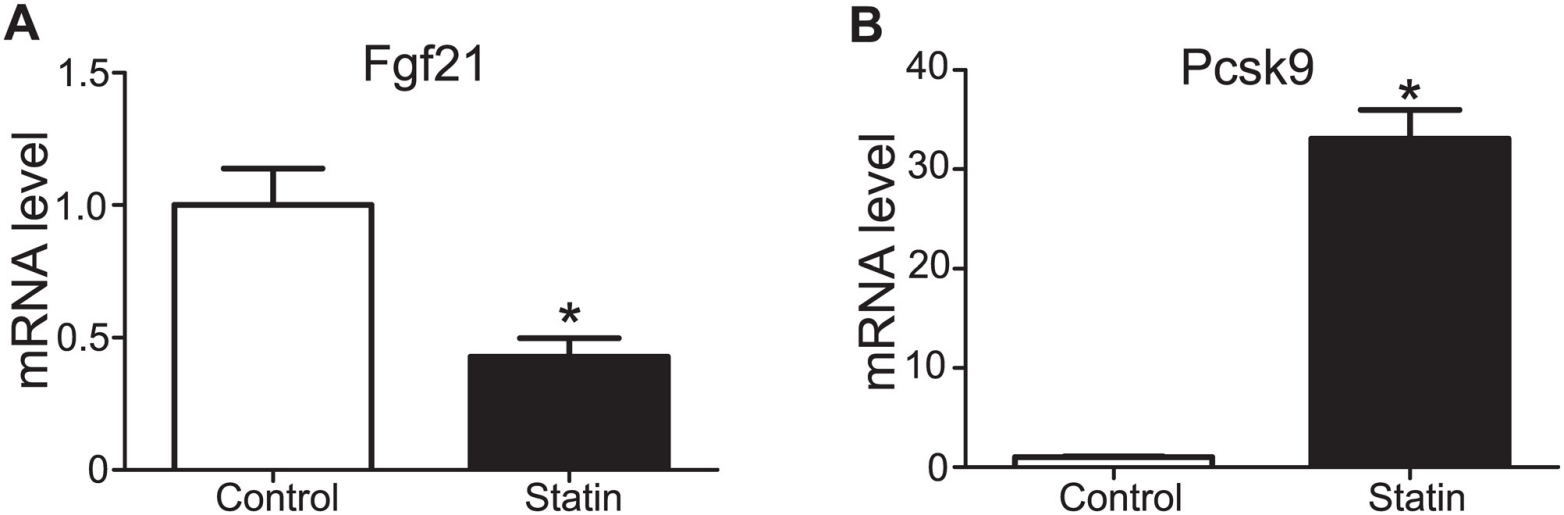
Fgf21 mRNA levels after intraperitoneal administration of simvastatin. Fgf21 (**A**) and Pcsk9 (**B**) mRNA levels in the liver of mice administered vehicle (control) or simvastatin intraperitoneally twice (20 and 12 hours before sacrifice). Data are presented as the mean ± SEM, n = 10 per treatment. *P<0.05 compared with control.

As chronic (1 week) administration of 0.1% w/w simvastatin in the chow led to weight loss, we wanted to eliminate the possibility that this effect influenced Fgf21 expression. To this end, we proceeded with a short-term (1 day) administration of activated simvastatin intraperitoneally during which no difference in body weight occurred. As presented in Fig 3A, IP administration of simvastatin reduced Fgf21 levels by 58%. mRNA levels of the positive control gene *Pcsk9* increased 33-fold, a finding that confirmed the efficacy of simvastatin treatment (Fig 3B).

### Simvastatin Lowered Hepatic Fgf21 Levels in a Cell Autonomous Manner

**Fig 4:**
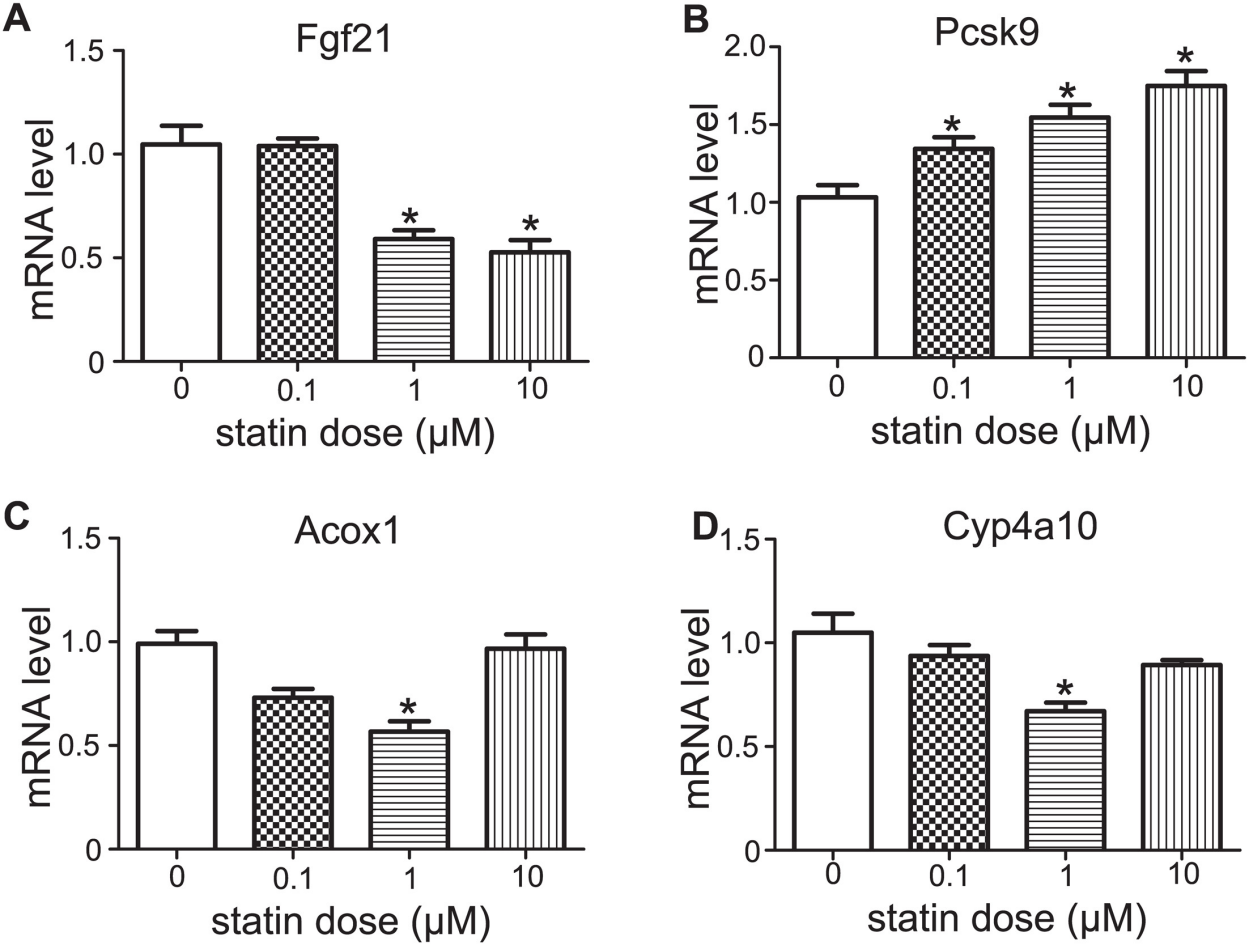
mRNA levels of Fgf21 and relevant genes in mouse primary hepatocytes after simvastatin treatment. mRNA levels of Fgf21 (**A**), Pcsk9 (**B**), Acox1 (**C**), Cyp4a10 (**D**) in primary hepatocytes treated with vehicle or simvastatin (3 different doses) for 12 hours. Data are presented as the means ±SEM from 3 individual experiments, each of which included 3 technical replicates. *P<0.05 (compared with vehicle treatment).

To eliminate the possibility that the Fgf21 mRNA levels are reduced during the primary mouse hepatocytes experiments, we assessed the Fgf21 levels 4 hours after plating (the time we allowed the primary hepatocytes to attach) until 28 hours post-plating. As shown in S1 Fig the mRNA levels of Fgf21 are relatively stable throughout this period. To determine whether the effect of simvastatin on hepatic Fgf21 levels is direct or indirect, mouse primary hepatocytes were treated with increasing doses of activated simvastatin as presented in Fig 4. Simvastatin was effective at all doses as indicated by the induction of the positive control gene *Pcsk9* (Fig 4B). Consistent with the *in vivo* effect of simvastatin on Fgf21 mRNA levels, cells treated with 1 and 10 μM of simvastatin exhibited decreased Fgf21 expression (Fig 4A). These findings support the possibility that the effect of simvastatin on Fgf21 is cell autonomous. The levels of Ppparα target genes was also examined. Acox1 and Cyp4a10 showed almost 50% decrease at the dose of 1μM (Fig 4C and 4D). Expression of Cyp7a1was too low to be detected accurately.

### Neither SREBP-2 nor miR-33 Overexpression Can Mimic the Simvastatin-Induced Reduction in Fgf21 Levels

**Fig 5:**
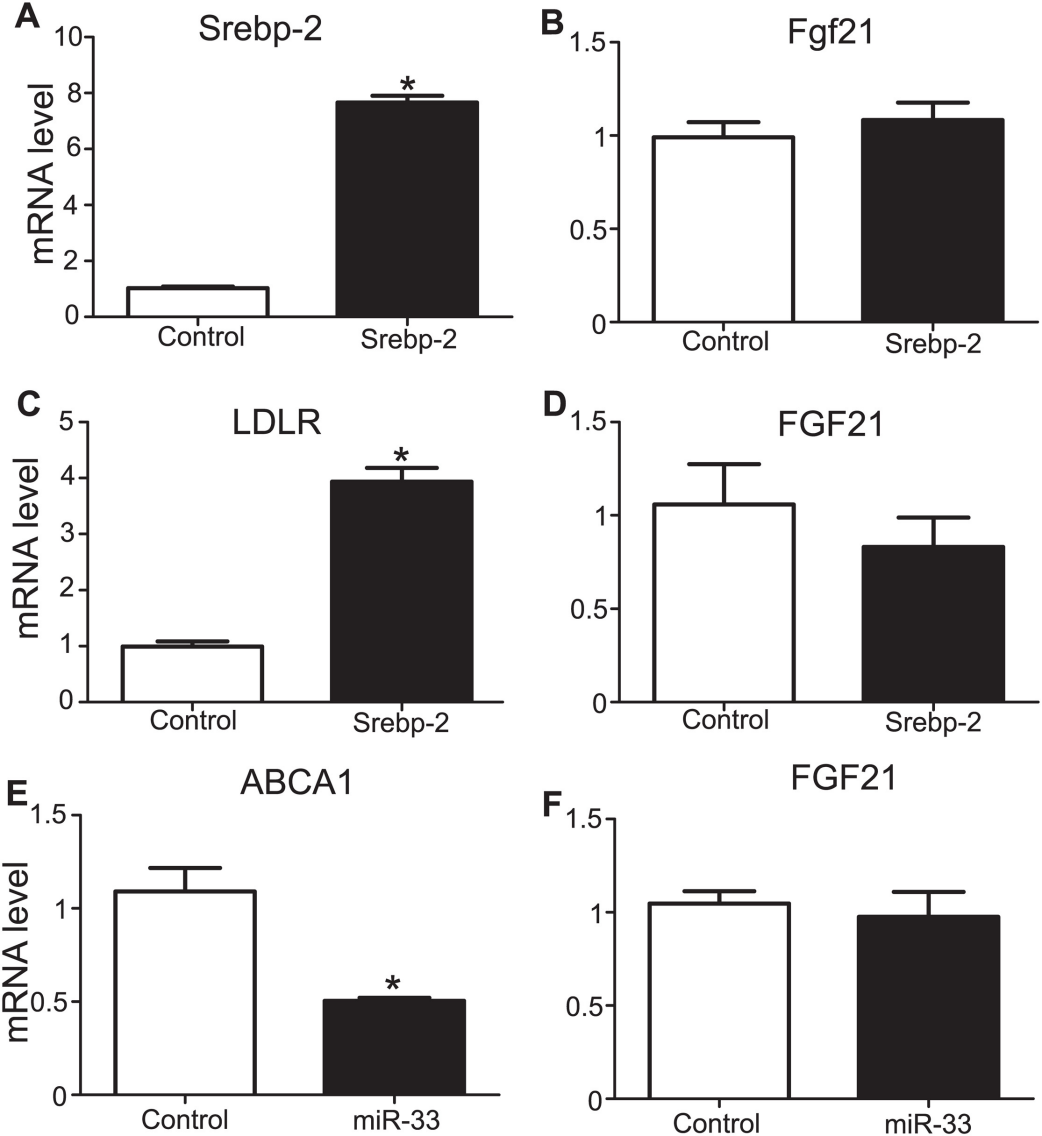
mRNA levels of Fgf21 in primary hepatocytes and HepG2 cells after overexpression of Srebp-2 or miR-33. Srebp-2 (**A**) and Fgf21 (**B**) mRNA levels in primary hepatocytes after overexpression of Srebp-2. Data are presented as the means ±SEM from 3 individual experiments, each of which included 3 technical replicates. *P<0.05 (compared with empty vector transfection). LDLR (**C**) and FGF21 (**D**) mRNA levels in HepG2 cells after overexpression of Srebp-2. Data are presented as the means ±SEM from 6 individual experiments, each of which included 3 technical replicates. *P<0.05 (compared with empty vector transfection). ABCA1 (**E**) and FGF21 (**F**) mRNA levels in HepG2 cells after overexpression of miR-33. Data are presented as the means ±SEM from 3 individual experiments, each of which included 3 technical replicates. *P<0.05 (compared with empty vector transfection).

As statins activate SREBP-2 we first evaluated if the overexpression of nSREBP-2 exerted the same effect on Fgf21 as simvastatin. To test this hypothesis, mouse primary hepatocytes were transfected with pcDNA3.1-2xFLAG-SREBP-2 and the increased expression of Srebp-2 (Fig 5A) did not seem to affect the Fgf21 expression levels (Fig 5B). HepG2 cells were also transfected with the SREBP-2 expressing plasmid and although this overexpression led to induction of LDLR (Fig 5C) as expected, no difference in FGF21 expression levels was seen (Fig 5D). In an analogous experiment, HepG2 cells were transfected with miR-33 adenoviral particles. As expected, the expression of the ATP-binding cassette transporter member 1 (ABCA1), a known target of miR-33, was reduced (Fig 5E). However, similar to nSREBP-2 overexpression, miR-33 overexpression did not significantly affect FGF21 expression (Fig 5F). It should be noted here that the relative expression level of FGF21 in HepG2 was lower compared to the mouse primary hepatocytes, increasing the standard error, that’s why some of the HepG2 experiments were repeated 6 times, as noted in the Fig 5 legend.

## Discussion

In this work, we demonstrated that Fgf21 expression decreased in the liver and serum of mice treated with simvastatin after an overnight fast and in primary hepatocytes treated with simvastatin. Furthermore, this effect tended to be dose-dependent and cell autonomous. These results were slightly unexpected given that our initial hypothesis proposed that statins induce Fgf21 by inducing mitochondrial and ER stress, processes which have been linked to Fgf21 activation[19–21, 58].

In an effort to determine a potential mechanism that mediates the Fgf21 lowering effect of statins, SREBP-2 was overexpressed in primary mouse hepatocytes and HepG2 cells, and miR-33 was overexpressed in HepG2 cells as both SREBP-2 and miR-33 are known to be upregulated by statins. Neither SREBP-2 nor miR-33 demonstrated an effect on FGF21 expression under the experimental conditions tested, although this cannot be ruled out completely as we have not used the relevant transgenic mouse models.

Available data regarding the potential association of Fgf21 and cholesterol metabolism are limited. Previous studies have shown that administration of Fgf21 can lower triglyceride levels in genetic models of obesity [59], can reduce ceramide levels in obese animals [60] and attenuate hyperglycemia in diabetic mice [61]. In human adipocytes, FGF21 has been shown to attenuate lipolysis [62] and an FGF21 analog has been demonstrated to ameliorate dyslipidemia in human subjects[63]. In mice deficient for Gp78, a ubiquitin ligase that mediates the degradation of HMG-CoA reductase, nuclear Srebp was downregulated, and Fgf21 levels were simultaneously upregulated[64]. Another study demonstrated that overexpressing Srebp-1c in HepG2 cells reduced the expression of Fgf21 at the mRNA level [65]. Based on these findings we tested if another member of the SREBP family, SREBP-2, which is primarily activated by statins, would have the same effect. However, as previously demonstrated, SREBP-2 was unable to reproduce the effect of simvastatin on Fgf21 expression.

Nevertheless, other potential mechanisms might underlie the simvastatin-induced downregulation of Fgf21. Specifically, Pparα, one of the primary inducers of Fgf21, might be associated with this effect. However, the existing literature does not support this speculation. Statins activate Pparα by inhibiting the Rho-signaling pathway [66], and a recent paper demonstrated that simvastatin is a Pparα ligand [67]. Our data cannot support or exclude the possibility that the lowering effect of simvastatin is mediated by Ppara.

Alternatively, the reduction in Fgf21 levels induced by statin treatment might potentially be mediated by a mechanism involving the mammalian target of rapamycin (mTOR) pathway. It has been reported that statins impair activation of mTOR by interfering with the prenylation of Rheb in vascular smooth muscle cells [68], and the activation of mTOR can induce Fgf21 in the liver by inducing amino acid deprivation [69]. A similar effect has been observed in muscle tissue [70].

As muscle tissue is able to express and secrete Fgf21 under conditions of stress, such as in mitochondrial myopathies in humans[71] and in mice[72], we evaluated the Fgf21 expression in the muscle of simvastatin-treated and control mice. However, the expression of Fgf21 mRNA was barely detectable in either group of mice (data not shown). Thus, no solid conclusions about the effect of simvastatin on Fgf21 expression in muscle tissue can be drawn from this study.

Regardless of the underlying mechanism, the observation that simvastatin reduces Fgf21 expression is interesting as it may uncover potential metabolic effects not only in mice but also in humans in studies that should follow. Fgf21 exerts beneficial effects on glucose, lipid and energy metabolism in mice and humans[73, 74]. The modulation of FGF21 expression by factors such as fructose[75] and protein restriction [76] in humans has instigated further investigation of the FGF21 modulating pathways. Therefore, understanding the pathways and drugs that can modulate the FGF21 expression is of potential importance to human metabolic diseases.

## Supporting Information

S1 Dataset Minimum dataset. This xlsx file contains a minimum dataset for the western blots, the ELISA and the qPCR after intraperitoneal statin administration of this paper.
(XLSX)

S1 Fig Fgf21 mRNA levels in primary hepatocytes up to 28 hours post-plating. Data are presented as the means ±SEM from 3 individual experiments, each of which included 3 technical replicates.
(PDF)
