# Supplementary material for: Hepatic Fgf21 Expression Is Repressed after Simvastatin Treatment in Mice
Source: PLoS One. 2016 Sep 1;11(9):e0162024. doi: 10.1371/journal.pone.0162024 (PMC5008788; doi:10.1371/journal.pone.0162024)
Supplement: S1 Fig — Data are presented as the means ±SEM from 3 individual experiments, each of which included 3 technical replicates. (PDF) [file pone.0162024.s002.pdf]

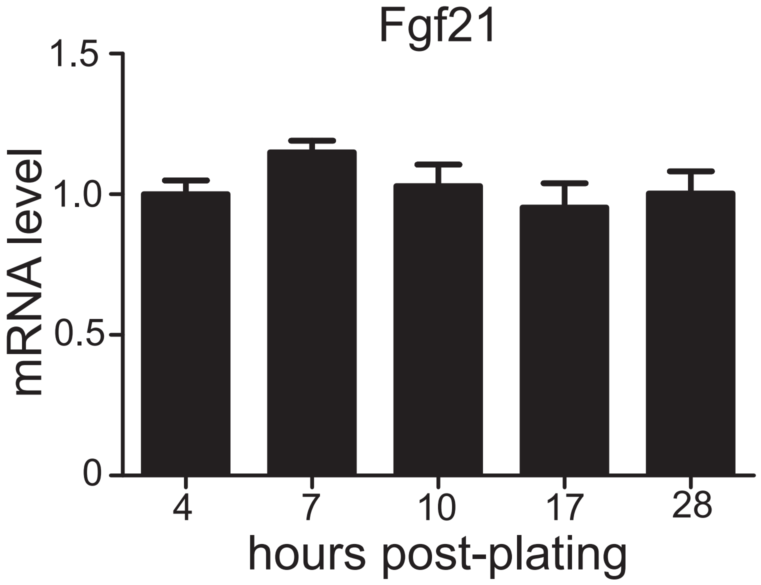

Fgf21
1.5
1.0
0.5
0
mRNA level
4
7
10
17
28
hours post-plating

Supplementary figure 1. Fgf21 mRNA levels 4, 7, 10, 17 and 28 hours post-plating of primary mouse hepatocytes. Data show mean ± SEM. Experiment was repeated 3 times with 3 technical replicates each time.
